# Il-6 Serum Levels and Production Is Related to an Altered Immune Response in Polycystic Ovary Syndrome Girls with Insulin Resistance

**DOI:** 10.1155/2011/389317

**Published:** 2011-03-20

**Authors:** Anna M. Fulghesu, Francesca Sanna, Sabrina Uda, Roberta Magnini, Elaine Portoghese, Barbara Batetta

**Affiliations:** ^1^Dipartimento Chirurgico Materno Infantile e di Scienze delle Immagini, Università di Cagliari, Via Ospedale, 09124 Cagliari, Italy; ^2^Dipartimento di Scienze e Tecnologie Biomediche, Università di Cagliari, Via Porcell 4, 09124 Cagliari, Italy

## Abstract

Polycystic ovarian syndrome (PCOS) is frequently characterized by obesity and metabolic diseases including hypertension, insulin resistance, and diabetes in adulthood, all leading to an increased risk of atherosclerosis. The present study aimed to evaluate serum and production of inflammatory markers in adolescent Sardinian PCOS. On the basis of HOMA findings, patients were divided into noninsulin resistant (NIR) and insulin resistant (IR), and were weight- and age-matched with healthy girls. Inflammatory cytokines (TNF-**α**, IL-6, Il-10, TGF-**β**) and lipokines (leptin, adiponectin), the reactant hs-CRP, and *in vitro* inflammatory lympho-monocyte response to microbial stimulus were evaluated. In healthy and PCOS subjects, leptin and hs-CRP were correlated with BMI, whereas adiponectin was significantly reduced in all PCOS groups. Although cytokines were similar in all groups, Interleukin-6 (IL-6) was significantly higher in IR PCOS. Moreover, in the latter group lipopolysaccharide-activated monocytes secreted significantly higher levels of IL-6 compared to NIR and control subjects. To conclude, IR PCOS displayed increased IL-6 serum levels and higher secretion in LPS-activated monocytes, whilst revealing no differences for other inflammatory cytokines. These results suggest that in PCOS patients an altered immune response to inflammatory stimuli is present in IR, likely contributing towards determining onset of a low grade inflammation.

## 1. Introduction

Polycystic Ovarian Syndrome (PCOS) is a complex disease characterized by hyperandrogenism and chronic anovulation [1]. The detection of traditional cardiovascular (CV) risk factors, that is, insulin resistance, diabetes, visceral obesity [2–6], and a state of low-grade inflammation [7–10], places these patients at a significantly higher risk for development of symptomatic atherosclerotic CV diseases. The presence of a cluster of metabolic and inflammatory factors has often been described in obese PCOS compared with age- and weight-matched controls. Higher serum levels of hs-CRP [11], IL-6 [12], and leptin have been reported, the latter being a proinflammatory molecule mainly produced by adipose tissue [13–15]. The latter alterations were all related to BMI, mainly increased visceral adipose tissue, frequently observed in these patients and which in turn correlates with insulin resistance [8, 16]. Conversely, adiponectin, an anti-inflammatory lipokine was found to be decreased in all PCOS patients [17, 18]. Although the majority of related articles focus on CV risk in adult PCOS, with this aspect currently being studied also in young PCOS patients, the various inflammatory markers are frequently investigated in separate studies [2, 9, 19, 20]. The aim of the present paper was to evaluate the most frequently investigated inflammatory cytokines during the early stages of reproductive life in a Sardinian PCOS population, both with or without insulin resistance. Indeed, the study evaluated cytokine production following microbial and mitogenic stimuli in lymphomonocytes from healthy and PCOS subjects, as these molecules are primarily of immunological origin. 

## 2. Subjects and Methods

### 2.1. Recruitment and Characterization of Study Participants

From January 2007, 44 young girls aged 15–23 affected by PCOS were recruited at the Adolescent Centre for Gynecological Diseases, Department of Obstetrics and Gynecology, “San Giovanni di Dio” Hospital, University of Cagliari. The diagnosis of PCOS was made according to Rotterdam criteria [21] in the presence of at least two of the following: (1) oligomenorrhea (40 subjects) and/or anovulation (4 subjects), (2) hyperandrogenism (clinical and/or biochemical); (3) polycystic ovaries with the exclusion of other etiologies. All subjects had previously been screened to exclude other causes of hyperandrogenism, such as androgen-secreting tumors and congenital adrenal hyperplasia (tested by evaluation of 17-4 hydroxyprogesterone), and for euthyroid status, hyperprolactinemia, diabetes, hypertension, and other cardiovascular diseases. Over the three months preceding the study no subject had been on hormonal contraceptives, other medications or diet which could have affected lipid profile, carbohydrate metabolism, and insulin levels. No subject smoked or consumed alcohol. All study participants were asked to refrain from intense physical activity. 

Fifty-five healthy young female volunteers with regular menstrual cycles, matched for BMI and with no clinical or biochemical signs of hyperandrogenism and ultrasound exclusion of PCO, took part as a control group. Subjects were all free from medication at the time of the study. No participants manifested or referred recent clinical infections. Investigations were conducted between days 5 and 8 from the last menstrual cycle. The latter was induced in 4 patients with amenorrhoea by administering progestinic drugs.

### 2.2. Measures and Analysis

Hormonal study (after 12 hours fasting) included a baseline plasma determination of androstenedione (A), testosterone (T), and prolactin (PRL). LH, FSH, estradiol (E2), dehydroepiandrosteronesulphate (DHEAS); 17-hydroxyprogesterone (17-OHP), and sex hormone binding globulin (SHBG) levels were also assayed but are not reported in the present paper. Plasma samples for hormone determination were maintained at −20°C until assay. All hormones were measured by RIA methods using commercial kits (Radim, Pomezia, and Ares Serono, Milan, Italy). Samples were immediately processed in a refrigerated centrifuge, and the sera obtained stored at −20°C until assay. The intra-assay and interassay coefficients of variation obtained were <9% for all hormones. Blood samples for insulin (Diagnostic systems laboratories, INC. (DSL), Webster, TX) and glycemia (glucosidase method, Beckman glucose analyzer, Fullertonn Ca) were measured during a 75 g oral glucose tolerance test (OGTT), at 0, 30, 60, 90, 120, and 180 min from glucose load. A normal glycemic response to OGTT was defined according to the criteria of the National Diabetes Data Group [22]. Insulin and glucose responses were expressed as area under the curve (AUC-180), calculated according to the trapezoidal rule [23]. The homeostatic index of IR (HOMA) was estimated considering fasting glucose (mmol/L) and insulin (*μ*U/mL) divided by a constant (Io × Go/22.5) [24]. To identify the prevalence of IR in young PCOS patients, HOMA values obtained from control group were pooled to establish upper control values using the mean +2 standard deviation as previously described [19, 24, 25]. On the basis of 95% confidence limits, HOMA test was considered normal when lower than 65.6. Body mass index (BMI) was calculated according to the following formula: weight in kilograms/(height in meters) 2. To ascertain whether metabolic state could be associated with a peculiar inflammatory pattern, patients were divided into two groups according to HOMA status: nonresistant (NIR) and insulin resistant (IR). Results were further evaluated considering all PCOS patients as a single group.

### 2.3. Cytokine Assay

A serum sample from each participant was stored at −80°C for determination of IL-1*β*, IL-6, TNF-*α*, Il-10, TGF-*β*, hs-CRP, leptin, and adiponectin. Cytokines were assessed using a sandwich ELISA test (Biosource, Nivelles, Belgium) as reported previously [26]. Absorbance at 450 nm for all cytokines was measured with a model 680 microplate reader (Bio-rad, Hercules, CA). A standard curve was prepared by plotting absorbance value of the standard cytokine versus the corresponding concentration (pg/mL or ng/mL). The range of the assay was 7.8–500 pg/mL for IL-1*β*, 0.16–10 pg/mL for IL-6, 15.6–1000 pg/mL for TNF-*α*, 31.2–2000 pg/mL for IL-10, 15.6–1000 pg/mL for leptin, 0.5–32 ng/mL for adiponectin, and 78–5000 pg/mL for CRP. The range of assay for cytokine cultures was 7.8–1000 pg/mL for IL-1*β*, 7.8–1000 pg/mL for IL-6, 7.8–1000 pg/mL for TNF-*α*, 31.2–2000 pg/mL for IL-10, and 62.5–4000 pg/mL TGF-*β*. In some experiments the secretion of monocyte cytokines into medium was evaluated using samples obtained from several patients and controls. Cells were removed by centrifugation of 400 g for 10 min and supernatants frozen at −20°C until assay. Cell protein content was measured by Bradford assay [27].

### 2.4. Cell Isolation and Culture

Informed consent for *in vitro *studies was obtained from 8 IR, 5 NIR, and 8 control subjects. Lymphomonocytes were separated from a 25 mL of whole heparinized blood. Each blood sample was diluted twice with RPMI 1640 before centrifugation on Ficoll-Hipaque (density 1.077; Sigma Chemical Co.) at 800 g for 20 min at room temperature. The interphase band, containing the mononuclear cells, was collected and cells purified as previously described [28]. Purity for platelets, red cells, and granulocytes was checked. Viability, monitored by trypan blue dye exclusion, exceeded 98% in all experiments. Lymphomononuclear cells, at a density of 3 × 106/mL, were cultured in RPMI 1640 medium supplemented with 2% heat-inactivated human AB serum, penicillin 100 U/mL, streptomycin 100 mg/mL and 2 mM L-glutamine, and were allowed to adhere at 37°C in a 5% CO_2_ atmosphere for 2 hours. After removal of nonadherent cells, 80–90% of the observed cells was represented by monocytes, as assessed by anti-CD-14b staining and flow cytometric analysis. Cells were washed twice with prewarmed medium and cultured with RPMI-1640 supplemented with heat inactivated 10% AB plasma. To avoid the effect of unknown inflammatory factors present in plasma from patients and/or volunteers, commercially obtained AB serum (Sigma-Aldrich, Milan, Italy) was used. Cells were subsequently treated according to experimental requirements as described later in this section. Adherent monocytes were activated with LPS (serotype 026:B26, 100 ng/mL per 24). 

On completion of experiments medium supernatants were stored at −70°C and cytokines measured concomitantly to avoid variations in assay conditions. In some experiments, the suspended lymphocytes were lectin-stimulated (PHA 10 ng) and utilized to evaluate proliferative and inflammatory response. An aliquot of cells was hydrolyzed with NaOH for protein determination. 

### 2.5. Cholesterol Esterification

Adherent cells were incubated for 4 hours in medium containing [14C]-oleate bound to bovine serum albumin (BSA). To prepare the oleate-BSA complex, 3.7 MBq of [14C]-oleic acid in ethanol (Dupont, NEN specific activity 2.035 GBq/mmol) was mixed with 1.4 mg of KOH and the ethanol evaporated. PBS (1.5 mL) without Ca^2+^ and Mg^+^ containing 4.24 mg of BSA (fatty acid free, Sigma) was added and the mixture shaken vigorously. This solution was added to each well at a final concentration of 74 KBq/mL. After incubation, cells were washed with ice-cold PBS and lipids extracted with acetone. Neutral lipids were separated by TLC as previously described [28, 29] and incorporation of [14C]-oleate into cholesterol esters was measured. An aliquot of cell hydrolysate was processed for protein content.

### 2.6. Statistical Analysis

Data were expressed as mean ± SEM, and differences between groups were evaluated by the Student's *t*-test. For systemic markers of inflammation, owing to the prominent skewness of distributions, data were expressed as median, interquartile and min-max ranges and differences between groups were evaluated using the nonparametric Mann-Whitney *U* test. Nonparametric correlations between variables were evaluated by the Kendall tau correlation coefficient. 

## 3. Results

### 3.1. Anthropometric and Hormonal Characteristics in PCOS Patients and Controls

The study was approved by the Ethical Committee of the University of Cagliari. Written informed consent was obtained from all subjects and/or their parents or legal guardian before participating in the study. In line with the presence of peripheral insulin resistance (HOMA > 65,6), PCOS subjects were divided into 2 subgroups: 19 insulin-resistant (IR) and 25 noninsulin-resistant (NIR) subjects. As shown in Table 1, BMI, AUC-I 180, and HOMA-7,16 were significantly higher in IR PCOS. As expected, androgen and testosterone plasma levels were increased in all PCOS patients (Table 2). Menstrual irregularities were present in 90% of PCOS patients. Glycemia was normal in all subjects (data not shown). No significant differences were found in plasma lipids (data not shown). In accordance with the four criteria established by Cook (30), no subjects were affected by metabolic syndrome.

### 3.2. Systemic Markers of Inflammation

**Figure 1:**
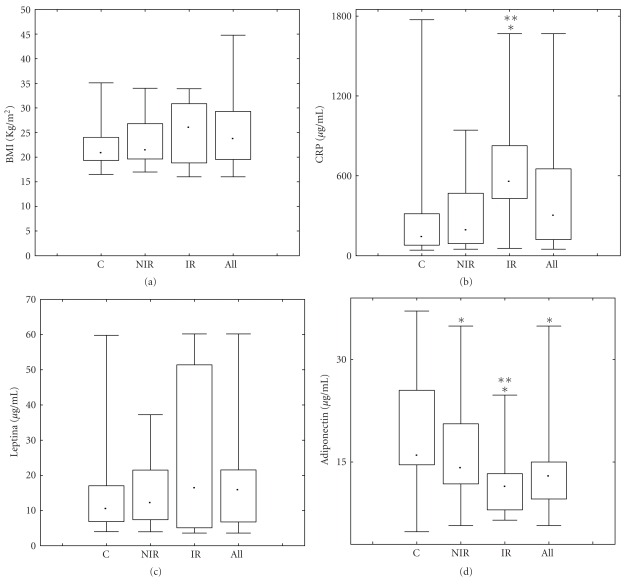
Leptin, BMI, adiponectin, and CRP in PCOS and control girls in controls (c), noninsulin resistant (NIR), and (IR) girls. Due to the strongly asymmetric distribution of data, the graph shows the median(middle bar), interquartile ranges (boxes), and min-max ranges (end bars). *P* <  .005 by Mann-Whitney *U* test.

**Figure 2:**
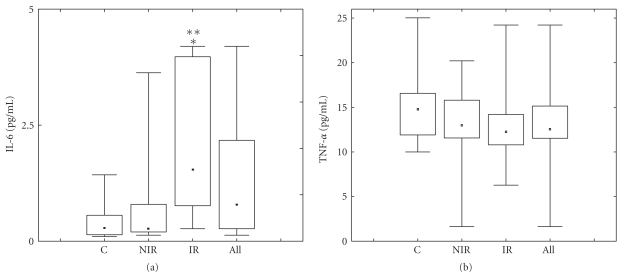
TNF-*α* and IL-6 levels in PCOS and control girls. Due to the strongly asymmetric distribution of data, the graph shows the median (middle bar), interquartile ranges (boxes), and min-max ranges (end bars). *P* <  .005 by Mann-Whitney *U* test.

As shown in Figure 1(a) leptin levels were higher in all PCOS groups, the higher values being found in IR patients (median: 16.30 pg/mL; min-max range: 3.6–120.00; *P* <  .05) on comparison of single groups to controls (median: 10.30 pg/mL; min-max range: 4.00–59.81), thus displaying a trend similar to that observed for BMI (Figure 1(b)). On the contrary, adiponectin (Figure 1(c)) was significantly decreased in all PCOS groups: IR (median: 11.40 ng/mL; min-max range: 6.50–24.80; *P* <  .05), NIR (median: 14.15 ng/mL; min-max range: 5.70–34.98; *P* <  .05), compared to control values (median: 15.80 ng/mL; min-max range: 4.80–37.10). CRP values, although revealing a trend similar to leptin and BMI, were significantly increased in IR (median 555 pg/mL, min-max range: 54.35–1668) compared to NIR (median 189.67 pg, min-max: 41.2–943) and control (median: 139,6 pg min-max range: 41,2–1772) groups (Figure 1(d)). No variation in TNF-*α* (Figure 2(a)) was observed in any group, and similar observations were made for IL-1*β*, Il-10, and TGF-*β* (data not shown). 

However, IL-6 (Figure 2(b)) was significantly increased in all groups, when compared to controls (median: 0.28 pg/mL; min-max range: 0.13–12), with a peak in IR (median: 2.79 pg/mL; min-max range: 0.56–12.00; *P* <  .001). When considering BMI and leptin (*r* = 0.6071; *P* <  .001), as well as BMI and adiponectin (*r* = −0.3387; *P* >  .002), both a positive and a negative correlation, respectively, was found in all subjects, independent of PCOS status. A correlation was detected between BMI and IL-6 in the control group (*r*2 = 0,64) but the range of IL-6 values was definitely lower than that observed in both NIR and IR groups. In addition, in both PCOS groups the correlation was very low (*r*2 = 0.004 and 0.16 NIR and IR, resp.). 

### 3.3. Production of Inflammatory Molecules in Lymphomonocytes

**Figure 3:**
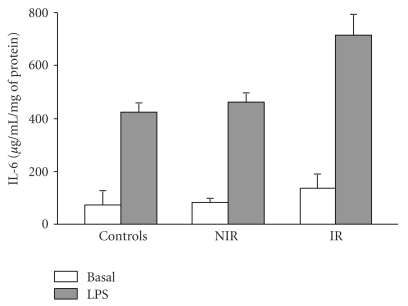
Il-6 secretion in LPS activated monocytes from PCOS and control girls. Each value represents mean ± SEM of 8 separated experiments. *LPS-activated monocytes from PCOS versus LPS- activated monocytes fromcontrols, *P* <  .05, by Student's *t*-test.

Inflammatory response was evaluated in lymphocytes and monocytes challenged with microbial stimulus. TNF-*α* and Il-*β*, as well as the anti-inflammatory cytokine Il-10 and TGF-*β* secretion in 24-hour LPS-activated monocytes, were similar in PCOS and control groups (data not shown). On the contrary, IL-6 production was remarkably and significantly higher in all LPS-treated monocytes from IR when compared to NIR and controls (Figure 3). 

Inflammatory cytokines and proliferative response evaluated in lymphocytes from the same subjects did not reveal significant differences between groups (data not shown). 

### 3.4. Cholesterol Esterification Did Not Vary in LPS-Treated Monocytes

**Figure 4:**
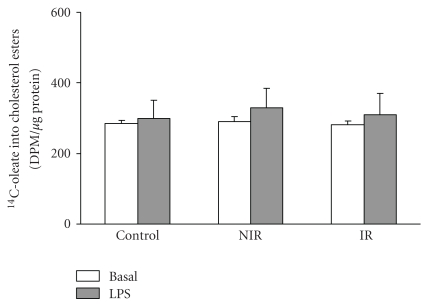
Cholesterol esterification in LPS-activated monocytes from PCOS and control girls. Each value represents mean ± SEM of 8 separated experiments.

As shown in Figure 4, LPS did not modify the rate of cholesterol esterification, the pathway leading to foam cell formation, hallmark of atherosclerosis, either in PCOS patients or controls.

## 4. Discussion

The present study demonstrates that the presence of a cluster of metabolic and inflammatory factors in insulin resistant PCOS girls is accompanied by an altered immune response to microbial stimulus, contributing to higher levels of serum IL-6. PCOS patients, frequently affected by visceral obesity and insulin-resistance [30, 31], feature an increased risk of developing atherosclerotic vascular disease later in life [2–7, 32–34]. A large body of reports published to date has focused on the evaluation in middle-aged females of metabolic, hormonal, and inflammatory factors accounting for low-grade inflammation leading to atherosclerosis [8–10, 35–40]. Although the majority of related articles focus on CV risk in adult PCOS, a condition of low-grade inflammation has also been described in adolescent girls [2, 20, 30, 40, 41]. In the PCOS population described in this study, BMI at times exceeded that obtained in controls, although no subjects examined developed diabetes. Accordingly, BMI and the prevalence of metabolic syndrome in the Italian female population has already been reported to be lower than figures obtained for their American counterpart [42, 43]. 

In the present study a cluster of molecules implicated in the onset of low-grade inflammation of the arterial wall was detected largely in adolescent girls developing insulin resistance. As reported [17, 18, 44], anti-inflammatory adiponectin alone was significantly lower in all PCOS patients examined, irrespective of BMI and insulin resistance, whereas in PCOS and normal healthy adults [11, 13, 14, 35], hs-CRP and leptin were positively correlated with BMI. The above molecules are likely related to the increased visceral adipose tissue often reported in the literature [8, 18, 45]. The increase revealed in waist to hip (W/H) ratio in PCOS subjects is a common observation [46–48] and is suggestive of a correlation with visceral adipose tissue, irrespective of BMI. 

Since visceral adipose tissue is associated with an increased production of a large number of functional molecules, it may be implicated, at least in part, in both insulin resistance and low-grade inflammation. A significant increase in serum proinflammatory cytokines was observed only for IL-6, featuring higher levels in all IR PCOS, independent of BMI. Although described by others [49] no increase of serum levels and production of TNF alfa was detected in our population. It is well acknowledged that IL-6 has been implicated in cardiovascular atherosclerotic risk, dyslipidemia, and hypertension. Moreover, it is a potent inducer of hepatic CRP [50], a molecule displaying increased levels in patients with severe atherosclerosis and following acute clinical events (myocardial infarction and cerebral ictus). It is a well-established fact that, in addition to visceral adipose tissue, activated lymphomonocytes produce large amounts of cytokines (for review see [51]). Investigations were thus undertaken to evaluate cytokine production in the latter cells, strongly involved in atherosclerotic plaque formation. Indeed, in the presence of an appropriate stimulus, these are the first cells recruited in the arterial wall [52]. Of all the pro- and anti-inflammatory cytokines evaluated, IL-6 production alone proved to be invariably higher in LPS-activated monocytes from IR subjects, whereas only 1 out of 5 NIR produced IL-6 comparably to IR cells. 

The results obtained suggest that an altered milieu due to insulin resistance may influence production of IL-6 in these cells. It may of course be argued that culture conditions do not reproduce the “in vivo” environment, however, in an attempt to better resemble ex vivo conditions, following separation, cells were immediately cultured in presence of LPS for 24 hours. Nevertheless, neither the susceptibility of monocytes to evolve into foam cells, nor mitogenic and inflammatory response to lectin in lymphocytes differed between IR and NIR PCOS and controls. Nevertheless, it remains to be seen whether potential differences may be manifested later in life or on the manifestation of a metabolic disease. Accordingly, an abnormal production of TNF alfa has been described in monocytes from diabetic PCOS [50]. 

To conclude, the results obtained in this study strongly suggest that hormonal and metabolic factors in young PCOS subjects are strongly associated with insulin resistance, and may concur to determine a condition of low-grade inflammation eliciting an overproduction of IL-6 monocytes in IR PCOS subjects following microbial inflammatory stimulus. This aspect should be carefully taken into consideration in view of recent evidence implicating infections as new atherosclerotic risk factors [53, 54]. Should proof of the latter be provided, further studies should be undertaken to investigate immune response in PCOS girls, particularly in view of the number of different microbial agents to which an individual is exposed during his or her lifetime. Finally, the results obtained suggest that high IL-6 in young PCOS subjects may also represent a signal of altered immune response that may enhance the identification of subjects featuring an increased risk of cardiovascular disease later in life. 

##  Funding

The study was funded by Fondazione Banco di Sardegna, Regione Autonoma della Sardegna and Nutrisearch Srl (Italy).

##  Conflict of Interests

There is no conflict of interest that could be perceived as prejudicing the impartiality of the research reported.

## Figures and Tables

**Table 1:** Anthropometric and metabolic characteristics of the study population (mean ± SD).

	Controls	PCOS	IR	NIR
*N*	55	44	19	25
Age (yr)	18.1 ± 3.1	19.1 ± 3.8	19.2 ± 3.8	19.1 ± 3.9
BMI (kg/m^2^)	24.8 ± 4.7*	25.1 ± 5.9	27.1 ± 7.2	23.2 ± 4.7
Oligomenorrhea %	0	90% (40 subjects)		
Amenorrhea %	0	3% (4 subjects)		
Waist (cm) (M ± ES)	81.2 ± 12.0	82.7 ± 16.1	88.6 ± 16.9	76.8 ± 15.4
Hips (cm) (M ± ES)	105.0 ± 10.0	102.6 ± 12.3	108.0 ± 12,9	97.3 ± 11.7
AUC-I 180	13,133 ± 7,115*	23,599 ± 9,998	24,961 ± 11,133	22,238 ± 8,864
HOMA- 7,16	46.7± 13.3*	65.2 ± 12.6	84.2 ± 14.3	46.8 ± 11.0

*P <  .05 versus IR patients.

**Table 2:** Hormonal characteristics of the study population (mean ± SD).

	Controls	PCOS	IR	NIR
*n*	55	44	19	25
T (nmol/L) (M ± ES)	0.4 ** ± ** 0.2*	0.6 ** ± ** 0.4	0.6 ** ± ** 0.4	0.7 ** ± ** 0.4
A (nmol/L) (M ± ES)	1.7 ** ± ** 0.8*	2.3 ** ± ** 1.0	2.2 ** ± ** 0.9	2.3 ** ± ** 1.1
PRL (ng/mL)	25.4 ** ± ** 8.1	23.1 ** ± ** 13.0	20.3 ** ± ** 10.6	26.0 ** ± ** 15.5
LH (IU/L) (M ± ES)	3.7 ** ± **1.5	3.9 ** ± ** 2.5	4.0 ** ± ** 2.1	3.9 ** ± ** 2.8
FSH (IU/L) (M ± ES)	6.1 ** ± ** 2.8	5.4 ** ± ** 1.6	5.7 ** ± ** 1.4	5.2 ** ± ** 1.8
E2 (pmol/L) (M ± ES)	17.6 ** ± ** 25.4	27.3 ** ± ** 26.5	22.6 ** ± ** 10.6	30.9 ** ± ** 33.7
DHEAS (*μ*mol/L) (M ± ES)	1.5 ** ± ** 0.6	2.2 ** ± ** 1.3	2.2 ** ± ** 1.3	2.2 ** ± ** 1.3
17OHP (ng/mL) (M ± ES)	1.2 ** ± ** 0.6	1.4 ** ± ** 1.8	1.1 ** ± ** 0.4	1.7 ** ± ** 2.4
SHBG (nmol/L) (M ± ES)	74.4 ** ± ** 34.7	66.6 ** ± ** 37.9	45.7 ** ± ** 24.7	81.4 ** ± ** 39.0

*P <  .05 versus IR and NIR.

## Abbreviations

AUC-180: Area under the curve for glucose above 180 mg/dL
BMI: Body mass index
HOMA-IR: Homeostasis model assessment of insulin resistance
hs-CRP: Highly sensitive Creactive protein
PCOS: Polycystic ovary syndrome.
